# Effects of Carbon, Nitrogen, Ambient pH and Light on Mycelial Growth, Sporulation, Sorbicillinoid Biosynthesis and Related Gene Expression in *Ustilaginoidea virens*

**DOI:** 10.3390/jof9040390

**Published:** 2023-03-23

**Authors:** Xuping Zhang, Xuwen Hou, Dan Xu, Mengyao Xue, Jiayin Zhang, Jiacheng Wang, Yonglin Yang, Daowan Lai, Ligang Zhou

**Affiliations:** Department of Plant Pathology, College of Plant Protection, China Agricultural University, Beijing 100193, China; zhangxuping5@cau.edu.cn (X.Z.); xwhou@cau.edu.cn (X.H.); cauxudan@cau.edu.cn (D.X.); mengyaoxue@cau.edu.cn (M.X.); jiayinzhang@cau.edu.cn (J.Z.); yonglinyang@cau.edu.cn (Y.Y.);

**Keywords:** sorbicillinoids, *Ustilaginoidea virens*, biosynthesis, carbon source, nitrogen source, ambient pH, light exposure, environmental factor, global transcription factor, pathway-specific regulator

## Abstract

Sorbicillinoids are a class of hexaketide metabolites produced by *Ustilaginoidea virens* (teleomorph: *Villosiclava virens*), an important fungal pathogen that causes a devastating rice disease. In this study, we investigated the effects of environmental factors, including carbon and nitrogen sources, ambient pH and light exposure, on mycelial growth, sporulation, as well as the accumulation of sorbicillinoids, and the expression of related genes involved in sorbicillinoid biosynthesis. It was found that the environmental factors had great influences on mycelial growth and sporulation of *U. virens*. Fructose and glucose, complex nitrogen sources, acidic conditions and light exposure were favorable for sorbicillinoid production. The relative transcript levels of sorbicillinoid biosynthesis genes were up-regulated when *U. virens* was separately treated with those environmental factors that favored sorbicillinoid production, indicating that sorbicillinoid biosynthesis was mainly regulated at the transcriptional level by different environmental factors. Two pathway-specific transcription factor genes, *UvSorR1* and *UvSorR2,* were found to participate in the regulation of sorbicillinoid biosynthesis. These results will provide useful information to better understand the regulation mechanisms of sorbicillinoid biosynthesis, and be conducive to develop effective means for controlling sorbicillinoid production in *U. virens*.

## 1. Introduction

*Ustilaginoidea virens* (teleomorph: *Villosiclava virens*) causes rice false smut (RFS), the most devastating disease in rice production [1,2,3]. RFS not only leads to considerable yield losses but also generates a diversity of mycotoxins that are poisonous to human beings, animals and the environment. The mycotoxins produced by *U. virens* mainly include ustiloxins, ustilaginoidins and sorbicillinoids, which show cytotoxic, antimicrobial and phytotoxic activities [4,5,6,7,8,9,10,11,12]. Sorbicillinoids are a family of polyketides, and typically contain a sorbyl side chain in their structures with highly oxygenated frameworks [13]. Some of them belong to the group of mycotoxins, which are toxic to plants, humans, animals and the environment [14,15]. At least 159 sorbicillinoids have been identified in terrestrial and marine fungi [13,14,15]. The structural identification [14,15], biological activities [10,16,17], biosynthetic pathways [18,19,20], and physiological functions [21,22] of fungal sorbicillinoids have been widely studied.

Different regulation levels of secondary metabolism include environmental stimuli, global regulators, specific regulators, signal transduction pathways, epigenetic regulations, and post-translational modifications, as well as the combination of those regulations; they form a complex and multi-level regulatory network in fungi [23,24,25,26].

Environmental conditions, such as carbon and nitrogen sources, temperature, ambient pH, and light, as well as the interactions among these factors in the natural environment, may regulate the biosynthesis of secondary metabolites (SMs) in many filamentous fungi [27,28,29,30,31,32,33,34,35,36,37,38]. Meanwhile, environmental cues modulate the expression of numerous global regulators. Most biosynthetic gene clusters (BGCs) for SMs have pathway-specific regulators (also called cluster-specific regulators, or specific transcription factors) that act directly on the other genes located within these clusters. The expression of these internal regulators also depends on global transcription factors [35,36].

Some studies about the regulation of sorbicillinoid biosynthesis have been reported in other fungal species, such as *Penicillium chrysogenum* [39,40], *P. dipodomyis* [41] and *Trichoderma reesei* [21,42,43,44,45,46,47,48,49]. However, no research has been reported about sorbicillinoid biosynthetic regulation in *U. virens*. The carbon source and light are two important environmental factors affecting sorbicillinoid production, through global regulation [21,39,44,45]. The global regulators, such as methyltransferase-like LaeA (LAE1) and carbon catabolite repressor Cre1 (CreA), positively regulated conidia formation and sorbicillinoid biosynthesis in *T. reesei* [42,44,48]. SorR1 (YPR1) acts as a transcriptional activator, while SorR2 (YPR2) controls the expression of SorR1. Both pathway-specific transcription factors, SorR1 and SorR2, involved in sorbicillinoid regulation likely have a carbon-source-dependent function of balancing carbon and secondary metabolism, and SorR2 is subject to light regulation [39,43,44,45]. In addition, sorbicillinoid biosynthesis could also be affected by epigenetic regulation and signal transduction pathways [46,47,48].

The present study aimed at investigating the effects of different carbon and nitrogen sources, ambient pH and light exposure on mycelial growth, sporulation and sorbicillinoid production in *U. virens*, as well as the transcription levels and expressions of *sor* BGC genes involved in sorbicillinoid biosynthetic pathway under these conditions. In addition, two pathway-specific regulator genes, *UvSoR1* and *UvSoR2,* were deleted to investigate their functions on sorbicillinoid biosynthesis.

## 2. Materials and Methods

### 2.1. Fungal Strains, Plasmids and Culture Conditions

The wild-type (WT) strain P1 and gene deletion mutants of *U. virens,* as well as the plasmids used in this study, are listed in Table S1. The fungal strains were cultured in 100 mL YTD medium (yeast extract, 1 g/L; tryptone, 1 g/L; glucose, 10 g/L) at 28 °C with 160 rpm for 7 days. The culture broth was filtered through Miracloth (Billerica, MA, USA), and the conidia were collected, followed by counting with a hemocytometer, and then they were diluted to a concentration of 1 × 10^6^ conidia/mL in sterile distilled water. As *U. virens* did not grow well on the synthetic media, such as Czapek–Dox medium, Base medium and Gause medium, PB solid medium (potato, 200 g/L; agar, 20 g/L) was found be suitable for sorbicillinoid production. So PB medium was selected as the basal medium, and was then used to investigate the effects of different environmental factors on mycelial growth, sporulation and sorbicillinoid production of *U. virens* [50].

### 2.2. Effects of Carbon and Nitrogen Sources, Ambient pH, and Light Exposure

Yeast extract powder and tryptone were purchased from Oxoid (Thermo Fisher Scientific, Waltham, MA, USA); yeast extract paste, beef extract, peptone and polypeptone were purchased from AOBOX Biotechnology Co. Ltd. (Beijing, China); dextrin and glucose were purchased from Xilong Scientific Co., Ltd. (Shantou, China); apple pectin, stachyose, malt extract, maize powder, and soybean powder were purchased from Macklin Biochemical Co., Ltd. (Shanghai, China). Other chemicals and culture media were purchased from Sinopharm Chemical Reagent Co., Ltd. (Shanghai, China).

For the carbon source assessment, the PB medium was supplemented with different carbon sources (i.e., soluble starch, apple pectin, fructose, glucose, sucrose, maltose, mannose, stachyose, dextrin, lactose, galactose, and xylose) at the final concentration of 10 g/L. The different basal media were employed for the determination of the effects of the following other environmental factors. They were used in independent experiments for the determination of the effects of different environmental factors. Only one variable was changed in each independent experiment.

For nitrogen source assessment, the PB medium was supplemented with glucose (10 g/L) as basal medium, with different nitrogen sources (i.e., yeast extract paste, yeast extract powder, beef extract, malt extract, maize powder, soybean powder, nutrient broth, peptone, polypeptone, tryptone, KNO_3_ and urea), at the final concentration of 10 g/L.

For the assessment of carbon and nitrogen sources, PB medium was used as the basal medium. In order to better evaluate the influences of environmental factors, we carried out the following pH and light experiments, based on the assessment results of the carbon and nitrogen sources.

To test the effects of ambient pH on mycelial growth, sporulation and sorbicillinoid production in *U. virens*, the pH values of PBGY medium (PB medium, supplemented with 10 g/L glucose and 10 g/L yeast extract paste) were amended to between 3.0 and 8.0, with either acid or alkali solution (1 M HCl and 1 M NaOH).

To determine *U. virens’s*’ response to light exposure, *U. virens* was grown on PBGB medium (PB medium, supplemented with 10 g/L glucose and 10 g/L beef extract) and was cultured in an LED light incubator at 28 °C, with different light treatments (DD, 24 h dark/day; LL, 24 h light/day; DL, 12 h dark–12 h light/day). Light was provided by conventional LED bulbs with 4000 Lux light intensity, and the color temperature (Kelvin) of the LED bulbs was 5000 K.

### 2.3. Mycelial Growth and Sporulation Assessments

To evaluate the effects of different carbon and nitrogen sources, ambient pH, and light exposure on mycelial growth and sporulation, the plates with different environmental factors were inoculated with 2 μL conidia suspension (1 × 10^6^ conidia/mL) of *U. virens,* and were cultivated at 28 °C in darkness. For growth assessment, the plates with mycelia were photographed on day 18 of cultivation, then the colony extension diameters were measured. For mycelia biomass measurement, 2 μL of conidia suspension (1 × 10^6^ conidia/mL) was cultivated on different medium plates, covered with a sheet of sterile cellophane at 28 °C, in darkness, for 18 days. Then, the mycelia were collected from the medium through cellophane separation, dried in an oven at 55 °C to a constant weight, and the dry weight was then determined. During the experiment, we also directly inoculated *U. virens* onto the agar plates supplemented with different carbon sources, without cellophane covering the agar medium. It was found that the growing status of the fungus was almost the same as that grown with cellophane. For sporulation assessment, a 100 μL conidia suspension (1 × 10^6^ conidia/mL) of *U. virens* was coated onto a 60 mm-diameter plate with different environmental factors, and cultured at 28 °C in darkness for 18 days. Two mycelial plugs with diameters of 10 mm were taken from each plate, then homogenized in 5 mL water containing 0.05% Tween 80, and filtered with Miracloth. The spore suspension was diluted to a suitable concentration. The conidia quantity of each plate was determined using a hemocytometer. The sporulation is presented as conidia number per square millimeter (conidia/mm^2^) of agar surface [51,52].

### 2.4. Sorbicillinoid Production Determination

The 28 day-old mycelia on the cellophane were separated from different media in plates, and dried at 55 °C in an oven to obtain the dry weight. Then, the dried mycelia and their medium were extracted with 10 mL analytical reagent-grade methanol in an ultrasonic bath three times (35 kHz, 30 min). After removal of the solid, by filtration, the filtrate was evaporated to dryness and re-dissolved in 1 mL of chromatographic grade methanol. Three main sorbicillinoids, including trichotetronine (also called bislongiquinolide), demethyltrichodimerol, and trichodimerol, were detected and quantified with the standards obtained from our previous study, which were identified according to their molecular weight, retention times and spectrometric data, with their structures shown in Figure S1A [11]. In terms of analyzing the contents and yields of major sorbicillinoids, HPLC analysis was performed by injecting 10 μL of the filtrate extract, with a microporous filter (pore size, 0.22 μm), into a Shimadzu instrument consisting of an SPD-M20A photodiode array detector (LC-20A, Shimadzu Corp., Tokyo, Japan), using an analytic C18 column (250 mm × 4.6 mm i.d., 5 μm; Phenomenex Inc., Torrance, CA, USA). The column temperature was set to 30 °C. The mobile phase was composed of methanol (mobile phase B), and water, containing 0.02% TFA (mobile phase A). A gradient elution program, eluting from 60% to 100% methanol over 40 min, was used; the flow rate was 1.0 mL/min and detection wavelength was 370 nm. Three main sorbicillinoids were quantified with the standard regression equations; the regression equations were *Y* = 1,138,395.3477 *X* − 3022.8909 (*R* = 0.9982) for trichotetronine, *Y* = 2,254,860.6801 *X* − 18,274.5152 (*R* = 0.9995) for demethyltrichodimerol, and *Y* = 4,854,544.1694 *X* + 29618.4711 (*R* = 0.9992) for trichodimerol, where *Y* is the peak area, *X* is the quality (μg) of the sample injected each time, and *R* is the correlation coefficient. Sorbicillinoid production was assessed by content (mg/g) and yield (mg/L). The sum of trichotetronine, demethyltrichodimerol and trichodimerol contents was considered to be the major sorbicillinoid content. The sum of three main sorbicillinoid yields was considered to be the major sorbicillinoid yield. Content (mg/g) was equal to total sorbicillinoids (mg), divided by mycelia dry weight (g), and yield (mg/L) was the total sorbicillinoids (mg) of per liter of culture broth, according to the method of Zong et al. [50].

### 2.5. RNA Preparation, RT–qPCR and Sorbicillinoid BGC Genes Analyses

A total of 2 μL of spore suspension (1 × 10^6^ conidia/mL) of *U. virens* was inoculated onto each plate, and covered with sterile cellophane at 28 °C for 10 days. Mycelia were harvested and stored at −80 °C for RNA extraction. All tips and mortars were autoclaved twice to prevent RNase contamination, and the mycelia were ground into powder, with liquid nitrogen, in a mortar, manually. According to the operation manual, total RNA was extracted from 100 mg of mycelial sample using Trizol reagent (TransGen Biotech, Beijing, China), in order to lyse cells and inhibit nuclease release. The concentration and purity were measured with an ultraviolet spectrophotometer, and RNA integrity was checked by agarose gel electrophoresis. The reverse transcription of RNA into cDNA was performed using the Fast Quant RT Kit (TransGen Biotech, Beijing, China). The specific primers were designed and chosen to analyze the transcript level of genes (Table S2). The reverse transcription–quantitative PCR (RT–qPCR) assays were performed using the UltraSYBR One Step RT–qPCR Kit (CWBIO, Beijing, China) and the QuantStudio^®^ 6 Flex System (BioRad, Hercules, CA, USA). RT–qPCR was carried out in a total volume of 20 μL, containing 10 μL of SYBR Premix Ex Taq™, 0.4 μL of each primer (10 μM), 0.4 μL of Lox ROX Reference Dye, 1 μL of cDNA, and 7.8 μL of RNase free-water. The transcript levels from different samples were normalized to that of *β-tubulin* gene to compensate for variations in the amount of cDNA input. The *β-Tubulin* gene, used as the reference gene in this study, was considered to be stable [53,54,55]. Three biological and three technical replicates performed for each sample. Each relative transcript level was calculated by using the 2^−ΔΔCT^ method, with 100% amplification efficiency and using suitable internal reference gene [56]. To normalize the date and aesthetics of the heatmap, the *UvSorA* gene, showing moderate transcriptional levels in our previous research, was selected as the control [22]. Additionally, one culture condition, with moderate transcriptional levels of *sor* BGC genes, was selected as the control group in each independent environmental factor experiment. The *UvSorA* gene was used as the control in carbon and nitrogen assays, respectively, with the cultures grown in the media supplemented with maltose and malt extract. In the ambient pH assay, the control was the *UvSorA* gene, with the cultures grown in pH 5.5 medium. In the light exposure assay, the control was the *UvSorA* gene, with the cultures treated with DL light exposure. The relative transcript level was determined by log2FC (fold change) between the normalized value of the target gene in each sample and the control sample gene. Then, the package pheatmap in the R programming language (accessed on 20 July 2022) was used to visualize the gene expression data (https://cran.microsoft.com/snapshot/2018-06-22/web/packages/pheatmap/pheatmap.pdf).

### 2.6. Gene Deletion and Phenotyping

All primers used to construct *UvSorR1* and *UvSorR2* knockout mutants are listed in Table S2. The gene deletion via the CRISPR/Cas9 system was performed according to the previous method [57,58]. The gene deletion cassette was constructed by upstream and downstream flanking sequences of the target gene, fused with the geneticin-resistance gene (*NeoR*). The gRNA spacers were cloned into the CRISPR-Cas9 vector (pCas9-tRp-gRNA). *U. virens* protoplasts were transformed by PEG-mediated transformation, and the mutants were selected from the medium containing 700 μg/mL of G418 (Sigma-Aldrich, St. Louis, MO, USA). Genomic DNA was extracted from the mycelia of transformants, by using a regular phenol–chloroform method, and correct gene replacements for upstream, downstream and target genes were confirmed by diagnostic PCR.

For mycelial growth and sporulation assessments, 2 μL of spore suspensions (1 × 10^6^ conidia /mL) from the WT strain and deletion mutants were inoculated on PDA medium (potato, 200 g/L; glucose, 20 g/L; agar, 20 g/L) at 28 °C for 18 days. Colony extension diameter, mycelia dry weight and spore concentration were measured. For chemical analyses, the WT strain and deletion mutants were grown on GYES medium (yeast extract, 10 g/L; glucose, 10 g/L; starch 10 g/L; NaCl, 5 g/L, CaCO_3_, 3 g/L) at 28 °C for 28 days. The MeOH extracts of the plates with hyphae were analyzed by HPLC.

### 2.7. Statistical Analysis

All experiments were designed with three independent biological replicates. Five replicates were performed for each treatment. Similar results were garnered for each biological experiment. Statistical analysis was carried out using SPSS version 17.0 (SPSS, Inc., Chicago, IL, USA), with a one-way analysis of variance (ANOVA) and Duncan’s multiple range test. Data are expressed as standard error (SE) of the mean, and differences at *p* < 0.05 were considered statistically significant.

## 3. Results

### 3.1. Effects of Carbon Sources on Mycelial Growth, Sporulation and Sorbicillinoid Production

**Figure 1:**
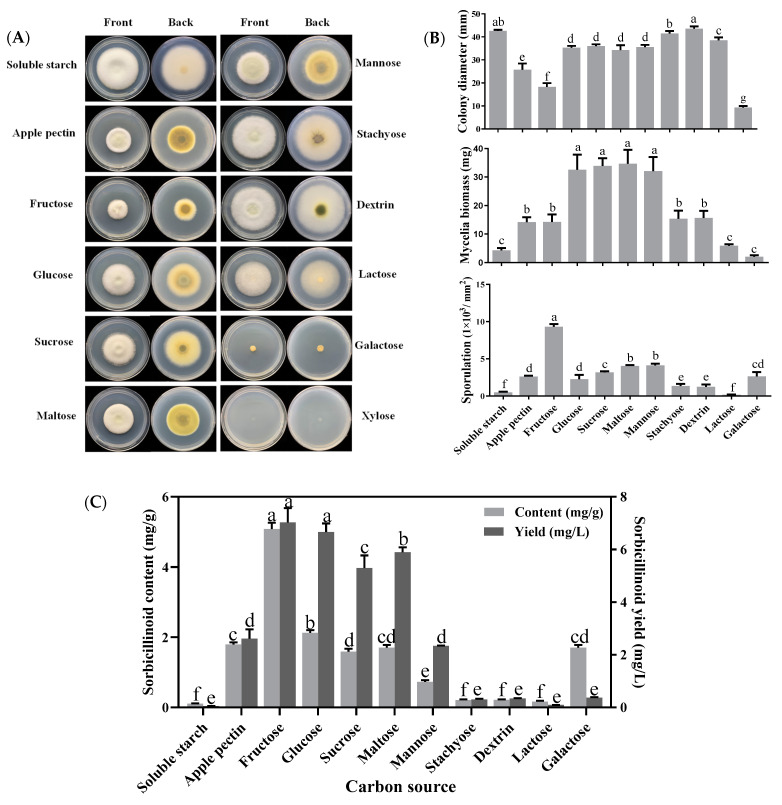
Effects of different carbon sources on colony extension, mycelial growth, sporulation and sorbicillinoid production of *U. virens*. (**A**) Colony morphology of *U. virens,* cultured for 18 days at 28 °C on PB medium, supplemented with different carbon sources; (**B**) Effects of different carbon sources on colony diameter, mycelial biomass and sporulation of *U. virens*; (**C**) Effects of different carbon sources on sorbicillinoid content (mg/g) and yield (mg/L). Bars represent standard deviations of the means. Different letters in each figure mean significant differences according to Duncan’s Multiple Range Test (*p* < 0.05).

Twelve different carbon sources were tested for their effects on mycelial growth, sporulation and sorbicillinoid production in *U. virens*. Except for the fact that *U. virens* could not grow on medium with xylose as the carbon source, the colony morphology and pigment accumulation varied when *U. virens* was cultured with other carbon sources (Figure 1A). Colony diameter and mycelial biomass were used to evaluate mycelial growth. An increase in mycelial radial expansion was observed when the medium was separately supplemented with dextrin, soluble starch, stachyose and lactose, but these carbon sources had less mycelial biomass accumulation than glucose, sucrose, maltose, and mannose. For sporulation, it was observed that the maximum sporulation (9.3 × 10^3^ conidia/mm^2^) of *U. virens* was observed when adding fructose, while minimum sporulation (2.0 × 10^2^ conidia/ mm^2^) was recorded when adding lactose (Figure 1B).

Sorbicillinoid production was also obviously influenced by the carbon source. Fructose and glucose significantly promoted sorbicillinoid production, followed by maltose, sucrose, apple pectin, mannose, and galactose. In contrast, low sorbicillinoid production was detected in the medium supplemented with dextrin, stachyose, lactose and soluble starch, respectively (Figure 1C). Except for galactose, the content and yield of trichotetronine were the highest, while the content and yield of demethyltrichodimerol were the lowest among the three tested sorbicillinoids, regarding the media supplemented with all tested carbon sources (Table S3).

### 3.2. Effects of Nitrogen Sources on Mycelial Growth, Sporulation and Sorbicillinoid Production

**Figure 2:**
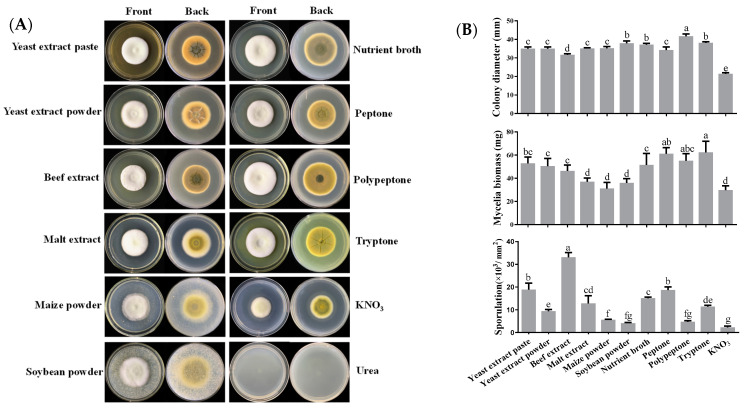

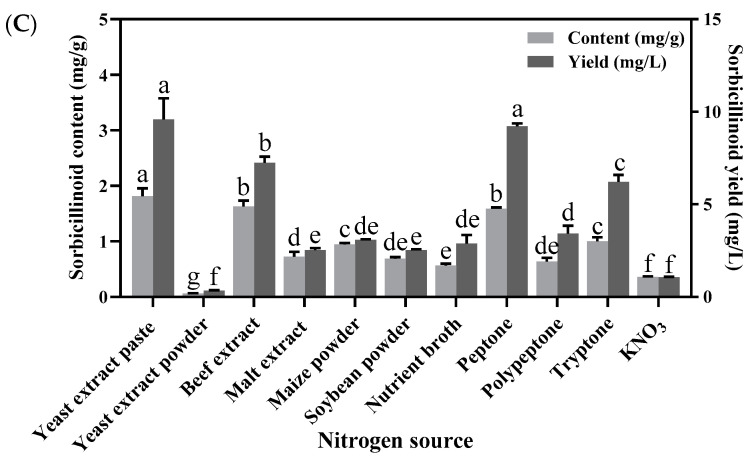
Effects of different nitrogen sources on colony extension, mycelial growth, sporulation and sorbicillinoid production of *U. virens*. (**A**) Colony morphology of *U. virens,* cultured for 18 days at 28 °C on PBG medium, supplemented with different nitrogen sources; (**B**) Effects of different nitrogen sources on colony diameter, mycelial biomass and sporulation of *U. virens*; (**C**) Effects of different nitrogen sources on sorbicillinoid content (mg/g) and yield (mg/L). Bars represent standard deviations of the means. Different letters mean significant differences according to Duncan’s Multiple Range Test (*p* < 0.05).

To investigate the effects of nitrogen sources on mycelial growth, sporulation and sorbicillinoid production, twelve different nitrogen sources, including one inorganic nitrogen source, and eleven organic nitrogen sources were tested in this study. The colony morphology and pigment accumulation were markedly influenced by the nitrogen source supplemented in the media, but *U. virens* could not grow in the media supplemented with urea (Figure 2A). Nitrogen sources affected the mycelial growth and sporulation of *U. virens*. Overall, organic nitrogen sources were more conducive to mycelial growth than potassium nitrate (KNO_3_). Peptone nitrogen sources (i.e., polypeptone, peptone and tryptone) promoted mycelial biomass accumulation. The maximum sporulation (3.32 × 10^4^ conidia/mm^2^) of *U. virens* was recorded in media supplemented with beef extract, followed by yeast extract paste, peptone, nutrient broth, malt extract and tryptone. Other nitrogen sources resulted in relatively less sporulation (Figure 2B).

Organic nitrogen sources resulted in increased sorbicillinoid production when compared to KNO_3_, except for yeast extract powder. Among ten complex nitrogen sources, yeast extract paste, peptone and beef extract were the most favorable nitrogen sources for sorbicillinoid production in *U. virens*. Tryptone was better for sorbicillinoid production, compared with polypeptone, maize powder, nutrient broth, malt extract, and soybean powder (Figure 2C). In addition, yeast extract paste and beef extract were more favorable for trichotetronine and demethylthyltrichodimerol production, while peptone was more favorable for trichodimerol production (Table S4).

### 3.3. Effects of Ambient pH on Mycelial Growth, Sporulation and Sorbicillinoid Production

**Figure 3:**
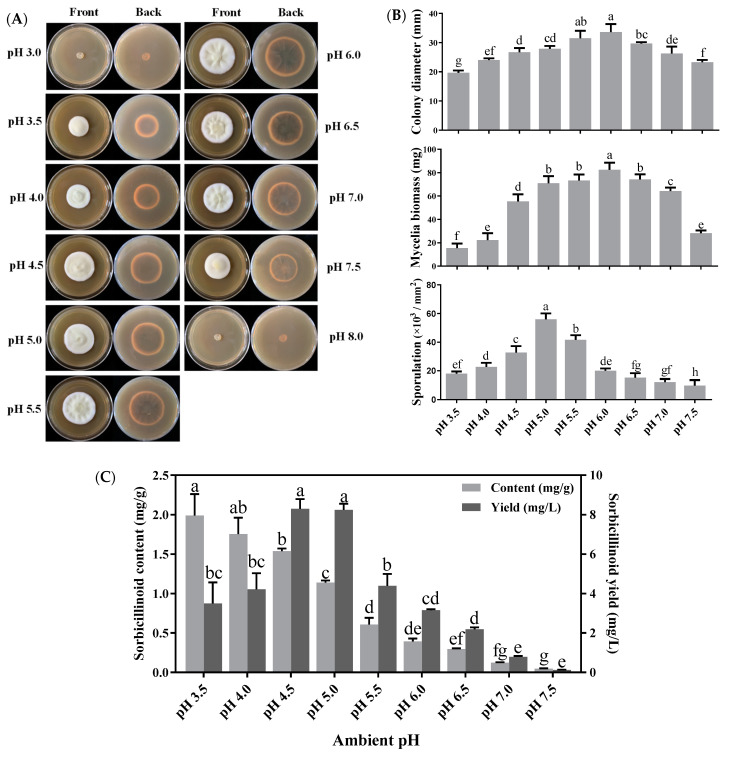
Effects of ambient pH on colony extension, mycelial growth, sporulation and sorbicillinoid production of *U. virens*. (**A**) Colony morphology of *U. virens,* cultured for 18 days at 28 °C on PBGY medium, with different ambient pH values; (**B**) Effects of ambient pH on colony diameter, mycelial biomass and sporulation of *U. virens*; (**C**) Effects of different ambient pH values on sorbicillinoid content (mg/g) and yield (mg/L). Bars represent standard deviations of the means. Different letters mean significant differences according to Duncan’s Multiple Range Test (*p* < 0.05).

The effects of ambient pH on mycelial growth, sporulation and sorbicillinoid production were established by culturing *U. virens* in PBGY medium, with pH values ranged from 3.0 to 8.0. *U. virens* had a broad pH range for mycelial growth and sporulation, but could not grow on the medium with pH values of 3.0 and 8.0 (Figure 3A). Mycelia grew well at pH 4.5 to 7.0, whereas mycelial growth was significantly inhibited at pH ≤ 4.0 and pH ≥ 7.5. The optimum pH value for mycelial growth and biomass accumulation in *U. virens* was pH 6.0. The highest sporulation (5.59 × 10^4^ conidia/mm^2^) was obtained at pH 5.0 (Figure 3B).

Ambient pH had a significant effect on sorbicillinoid production. The highest yield (8.3 mg/L) of sorbicillinoids was observed at pH 4.5, which was on par with that observed at pH 5.0. An acidic environment was more beneficial for sorbicillinoid accumulation in mycelia. The highest content (2.0 mg/g) of sorbicillinoids was observed at pH 3.5, and the content of sorbicillinoids was gradually decreased when pH was increased (Figure 3C). In addition, ambient pH also affected the composition of sorbicillinoids (Table S5). Trichotetronine was the major component in the sorbicillinoids when pH was 3.5, and distinctly reduced when the pH value was higher than 5.0. Trichodimerol was rarely detectable at pH 3.5, but its yield (2.2 mg/L) and content (0.4 mg/g) reached the maximum when the pH increased to 4.5 (Figure S1).

### 3.4. Effects of Light Exposure on Mycelial Growth, Sporulation and Sorbicillinoid Production

**Figure 4:**
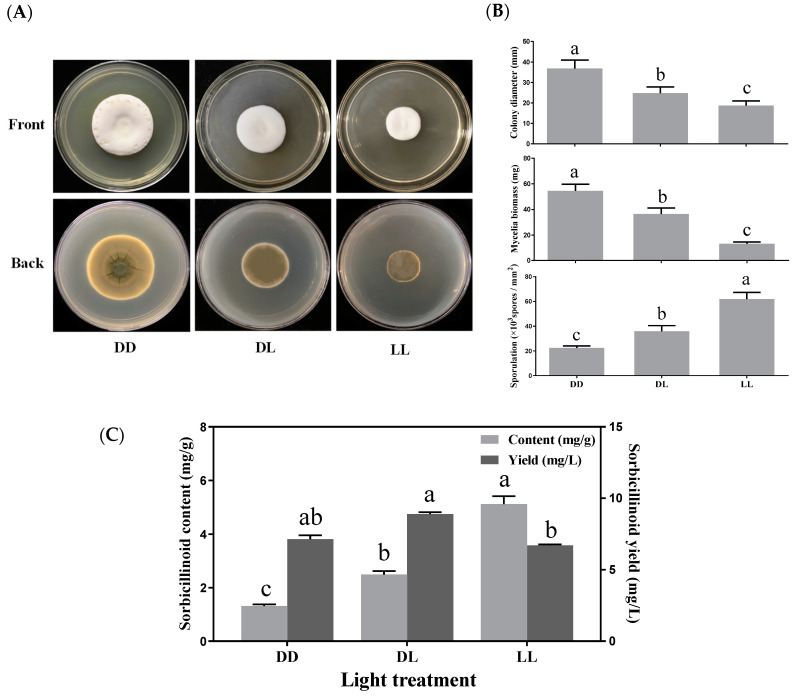
Effects of light exposure on colony extension, mycelial biomass, sporulation and sorbicillinoid production of *U. virens*. (**A**) Colony morphology of *U. virens,* cultured for 18 days at 28 °C, on PBGB medium with different light treatments; (**B**) Effects of light exposure on colony diameter, mycelial biomass and the sporulation of *U. virens*; (**C**) Effects of different light treatments (DD, 24 h dark/day; LL, 24 h light/day; DL, 12 h dark–12 h light/day) on sorbicillinoid content (mg/g) and yield (mg/L). Bars represented standard deviations of the means. Different letters in each figure mean significant differences according to Duncan’s Multiple Range Test (*p* < 0.05).

To investigate the effects of light exposure on mycelial growth, sporulation and sorbicillinoid production in *U. virens*, 24 h dark/day (DD), 24 h light/day (LL), and 12 h dark–12 h light/day (DL) periods were used for different light treatments. Light exposure influenced colony morphology and pigment accumulation (Figure 4A). When compared to darkness, the mycelial growth was reduced with light treatment, whereas sporulation was promoted by light exposure (Figure 4B).

Light exposure enhanced sorbicillinoid production, particularly regarding the content of sorbicillinoids in mycelia. Sorbicillinoid content was gradually increased with light exposure, and reached the maximum (5.12 mg/g) when the mycelia were treated with constant light (LL) (Figure 4C). The content of trichotetronine, demethylthyltrichodimerol and trichodimerol all displayed similar trends with light exposure. While the yields of trichotetronine (5.8 mg/L) and demethylthyltrichodimerol (1.1 mg/L) were highest with the DL treatment, the yield (1.4 mg/L) of trichodimerol was highest with the DD treatment (Table S6).

### 3.5. Relative Transcripts Levels of the Genes Involved in Sorbicillinoid Biosynthesis in Response to the Environmental Factors

**Figure 5:**
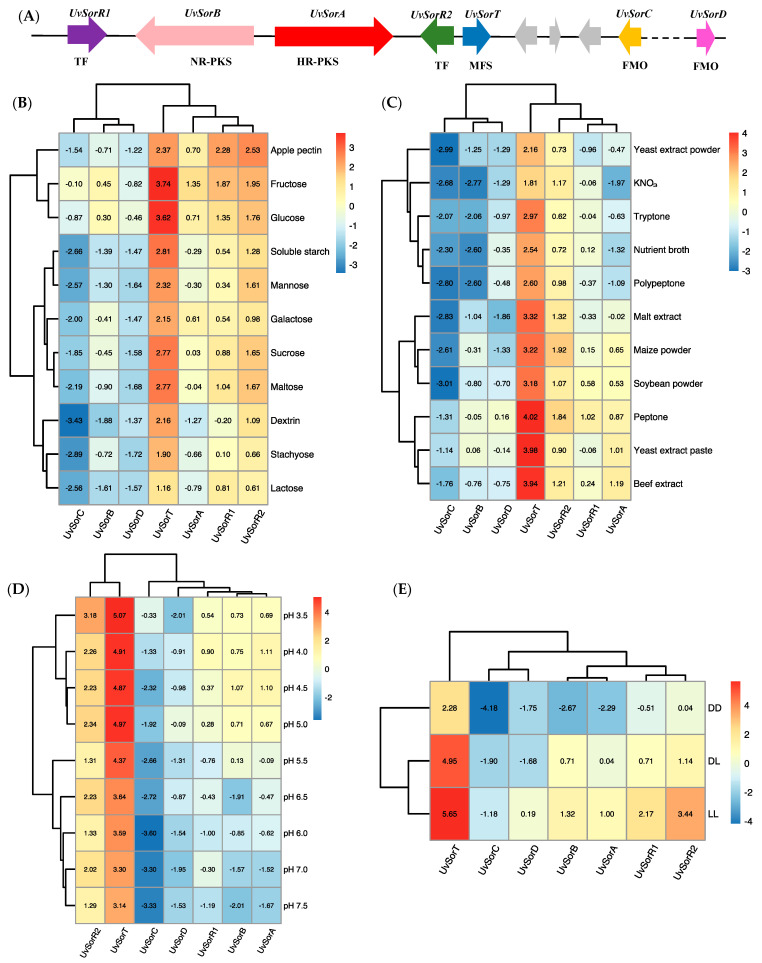
Heatmap based on the RT–qPCR analysis of the relative transcript levels of sorbicillinoid biosynthesis genes under different carbon, nitrogen sources, ambient pH, and light exposure. Relative transcript abundance was normalized to the level of *β-tubulin* with the 2^−ΔΔCt^ method. Numbers in the heatmap correspond to the log2 of fold change (FC) values of each gene, with *UvSorA* in the reference group (maltose, malt extract, pH 5.5 and DL treatment, respectively). Color scheme goes from red for upregulated genes to blue for down-regulated genes, when compared with the *UvSorA* gene in the reference group. (**A**) Schematic diagram of *sor* BGC. TF, transcription factor; NR-PKS, non-reducing polyketide synthase; HR-PKS, highly reducing polyketide synthase; MFS, major facilitator superfamily transporter; FMO, flavin-dependent monooxygenase; (**B**) The correlation heatmap between carbon sources and relative transcript level of sorbicillinoid biosynthesis genes; (**C**) The correlation heatmap between nitrogen sources and relative transcript level of sorbicillinoid biosynthesis genes; (**D**) The correlation heatmap between ambient pH and relative transcript level of sorbicillinoid biosynthesis genes; (**E**) The correlation heatmap between different light exposure treatments and relative transcript level of sorbicillinoid biosynthesis genes.

Seven conserved genes, *UvSorA*, *UvSorB*, *UvSorR1*, *UvSorR2*, *UvSorC*, *UvSorT* and *UvSorD,* participated in sorbicillinoid biosynthesis in *U. virens* [22] (Figure 5A). The RT–qPCR method was used to explore the transcript level patterns of these seven genes in response to different environmental factors. Overall, most of the transcript levels of these genes under various environmental factors were consistent with sorbicillinoid production in *U. virens* (Figure 5B–E).

When *U. virens* was cultured in the media with fructose, glucose, sucrose, and maltose as carbon sources, respectively for conducive sorbicillinoid production, the transcript levels of sorbicillinoid biosynthesis-related genes were up-regulated significantly when compared with low-sorbicillinoid-production carbon sources, such as soluble starch, stachyose, dextrin and lactose. The correlation heatmap of sorbicillinoid biosynthesis genes under different carbon sources shows that the high-transcript-level carbon sources fructose and glucose are clustered in one group. Two low-transcript-level carbon sources, stachyose and lactose, are clustered in another group. From gene cluster analysis, *UvSorB*, *UvSorC* and *UvSorD,* with the similar transcript patterns, are clustered in one group, while *UvSorR1*, *UvSorR2* and *UvSorA* are clustered in another group, and *UvSorT* is in a separated group (Figure 5B).

The transcript levels of sorbicillinoid biosynthesis genes were up-regulated significantly with the use of favorable nitrogen sources, including yeast extract paste, beef extract and peptone, and these nitrogen sources are clustered in one group. Malt extract, maize powder and soybean powder are clustered together, and the sorbicillinoid biosynthesis genes for this cluster present a moderate transcript level. Yeast extract powder, KNO_3_, tryptone, nutrient broth and polypeptone, as low-transcript-level nitrogen sources, belong to the same group (Figure 5C).

An acidic environment was more beneficial for high transcript levels of sorbicillinoid biosynthesis genes in *U. virens,* according to correlation heatmap of ambient pH. Interestingly, the relative transcript levels for most of the genes displayed a similar trend to sorbicillinoid content. Cluster analysis based on ambient pH shows that pH values 3.5–5.0 are grouped into one cluster, and pH values 5.5–7.5 are grouped into another cluster. By utilizing gene transcript profile cluster analysis, *UvSorT* and *UvSorR2* are in the same cluster, while the other genes are in another cluster (Figure 5D).

Light exposure with high expression level of sorbicillinoid biosynthesis genes enhanced sorbicillinoid content in *U. virens*. In particular, the expression level of the transcription factor gene *UvSorR2* in constant light (LL) was up-regulated 86-fold, relative to that observed in constant dark (DD). The heatmap cluster analysis shows that light exposure treatment clustered in one group, and sorbicillinoid biosynthesis genes are perfectly clustered according to their function. Two backbone genes (*UvSorA* and *UvSorB*), two tailoring genes (*UvSorC* and *UvSorD*), two regulatory genes (*UvSorR1* and *UvSorR2*) and one transporter gene (*UvSorT*) are clustered in different groups (Figure 5E).

On the whole, the expressions of these seven genes were increased in response to favorable carbon and nitrogen sources, an acidic pH and light exposure. However, the expressions of some genes did not change significantly in response to the environmental factors, or were even down-regulated (i.e., ambient pH). Therefore, the genes for which the fold changes were significant should be further analyzed.

### 3.6. Two Pathway-Specific Transcription Factor Genes UvSorR1 and UvSorR2 Involved in the Regulation of Sorbicillinoid Biosynthesis

#### 3.6.1. Deletion of Transcription Factor Genes UvSorR1 and UvSorR2

**Figure 6:**
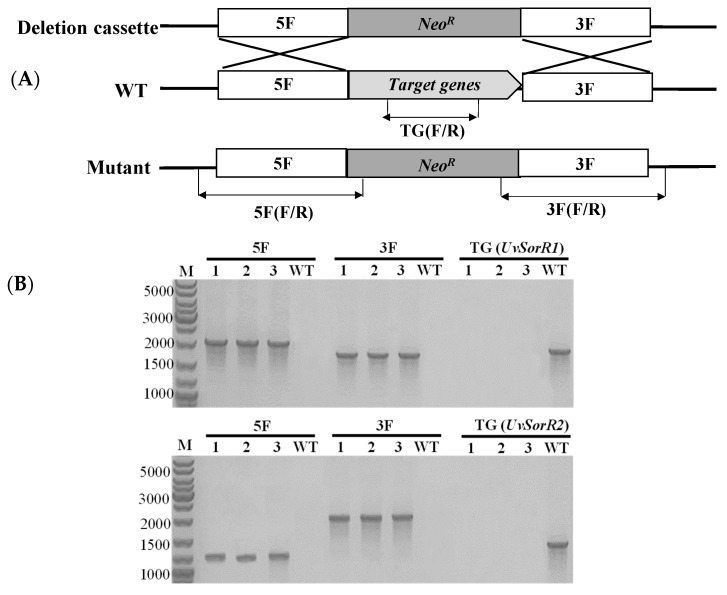
Generation of Δ*UvSorR1* and Δ*UvSorR2* deletion mutants in *U. virens*. (**A**) Schematic illustration of the disruption of the target gene (*UvSorR1* or *UvSorR2*). (**B**) In the *ΔUvSorR1* mutant, the upstream and downstream bands (2093 bp and 1719 bp) were only detected in the mutants (T1–T3), and the *UvSorR1* band (1882 bp) was only detected in the WT strain; in the Δ*UvSorR2* mutant, the upstream and downstream bands (1261 bp and 2042 bp) were only detected in the mutants (T1–T3), and the *UvSorR2* band (1616 bp) was only detected in the WT strain. Lane M: 1000 bp DNA Ladder.

Two transcription factors, *UvSorR1* and *UvSorR2,* in *sor* BGC were deleted with a geneticin-resistance (NeoR) cassette, in order to confirm their function in sorbicillinoid biosynthesis regulation. The deletion fragments, flanking approximately 1.0 kb upstream and downstream of *UvSorR1* or *UvSorR2* ORF regions, were fused partially with the geneticin-resistance gene. The gRNA spacers of *UvSorR1* and *UvSorR2* were cloned into the CRISPR-Cas9 vector (pCas9-tRp-gRNA). Both Δ*uvSorR1* and Δ*uvSorR2* mutants were generated by replacing the endogenous *UvSorR1* or *UvSorR2* ORF with the deletion cassette, via protoplast transformation with linear donor DNA fragments and the CRISPR construct. Genomic DNA was extracted from the transformants, and correct gene replacements were confirmed by diagnostic PCR (Figure 6). PCR assays using specific pairs of primers (Table S6) for the deletion of *UvSorR1* or *UvSorR2* were conducted, and then we further verified homologous recombination in both upstream and downstream flanking sequences in transformants.

#### 3.6.2. Regulation of UvSorR1 and UvSorR2 Involved in Sorbicillinoid Biosynthesis

**Figure 7:**
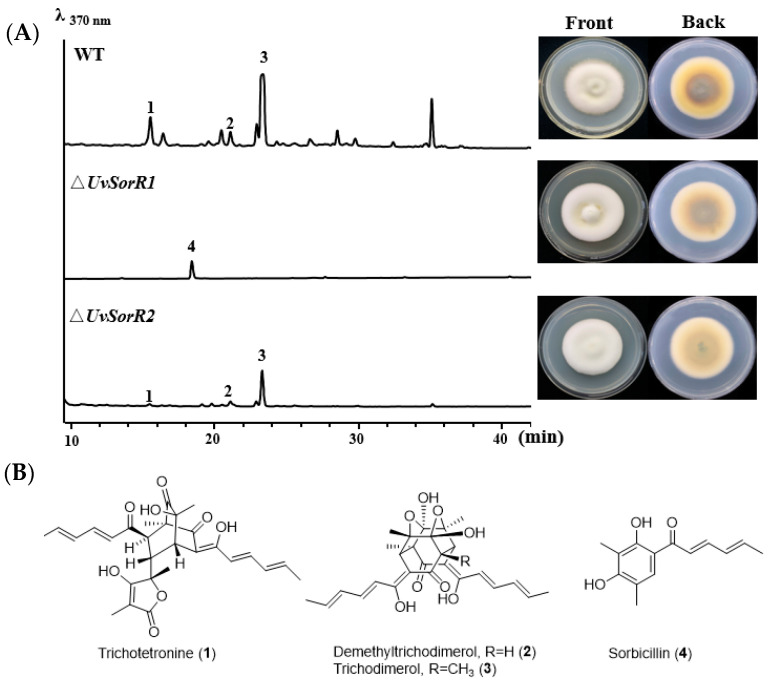
Colony morphologies and HPLC profiles of sorbicillinoids from the WT strain and two specific transcription factor deletion mutants. (**A**) HPLC profiles of the WT strain, Δ*UvSorR1* and Δ*UvSorR2* mutants detected with UV at 370 nm (left panel). All tested strains were cultured at 28 °C for 28 days on GYES medium for sorbicillinoid test. The colony morphologies was observed after 28 days of culture on the PDA plates (right panel). (**B**) Chemical structures of sorbicillinoids **1**–**4**.

There was no difference in mycelial growth and sporulation among Δ*UvSorR1* and Δ*UvSorR2* mutants and WT strain (Figure S2). In terms of sorbicillinoid synthesis of the wild-type (WT) strain*,* and the Δ*UvSorR1* and *ΔUvSorR2* mutants, the sorbicillinoids trichotetronine (**1**), demethylthyltrichodimerol (**2**) and trichodimerol (**3**) could be detected in the WT strain, but there was no dimeric sorbicillinoid detected in the Δ*UvSorR1* mutant; only a small amount of sorbicillin (**4**) could be detected when comparing it with the authentic compound. The sorbicillinoid production was drastically decreased in the Δ*UvSorR2* mutant. From the back views of the colonies, there was less pigment accumulation in Δ*UvSorR1* and Δ*UvSorR2* when compared with the WT strain (Figure 7). The gene deletion results showed that *UvSorR1* and *UvSorR2,* as pathway-specific transcription factors, indeed participate in the regulation of sorbicillinoid in *U. virens*.

#### 3.6.3. UvSorR1 and UvSorR2 Regulated Sorbicillinoid Biosynthesis at Transcriptional Level

**Figure 8:**
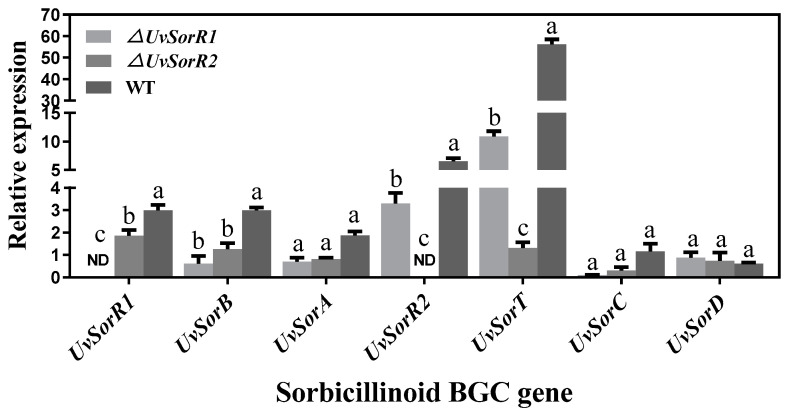
The relative transcript analysis of sorbicillinoid biosynthesis genes in the WT strain, as well as in Δ*UvSorR1* and Δ*UvSorR2* mutants. Different letters mean significant differences according to Duncan’s Multiple Range Test (*p* < 0.05).

The relative transcript levels of seven sorbicillinoid biosynthetic genes in the WT strain, as well as in the *ΔUvSorR1* and *ΔUvSorR2* mutants, were analyzed by qRT–PCR. The results reconfirm that *UvSorR1* and *ΔUvSorR2* had been successfully deleted according to the absence of fluorescent signal. When compared with the WT strain, the transcript levels of *sor* BGC genes were markedly down-regulated in Δ*UvSorR1* and Δ*UvSorR2,* except *UvSorD*, indicating that two pathway-specific regulators affected sorbicillinoid production by positively regulating the expression of *sor* BGC genes at the transcriptional level. Moreover, the expressions of *UvSorA* and *UvSorB,* as two PKS backbone genes, were still detected, but the relative expression level of the monooxygenase gene *UvSorC* was extremely low; a small amount of the backbone product sorbicillin was accumulated in Δ*UvSorR1* mutant (Figure 8).

## 4. Discussion

Environmental factors could affect mycotoxin biosynthesis [23,27,28,29,50]. Our results demonstrated that carbon and nitrogen sources, ambient pH and light exposure strongly influenced mycelial growth, sporulation and sorbicillinoid production in *U. virens*. A previous study reported that stachyose was a preferential carbon source utilized by *U. virens* [59]. However, our study showed that stachyose, soluble starch and dextrin only strongly increased colony diameter expansion, while their biomass accumulations were significantly less than sucrose, glucose, sucrose, maltose, and mannose, because of mycelium thinness. It was probable that, with the use of PB medium, rather than Czapek medium, as a basal medium, fructose could be utilized as a carbon source for *U. virens* mycelial growth, while xylose could not, which was inconsistent with previous reports [60]. Glucose was also a favorable carbon source for sorbicillinoid synthesis in *U. virens*, which was also observed in *T. reesei* [45,46]. Among the nitrogen sources, polypeptone, peptone and tryptone promoted mycelial biomass accumulation, while urea inhibited the mycelial growth of *U. virens,* which is consistent with the previous observations [60]. Yeast extract paste, peptone and beef extract could increase sporulation, and enhance sorbicillinoid production. *U. virens* adapted to pH values from 3.0 to 8.0, and the optimum pH value for mycelial growth and biomass accumulation was pH 6.0, which was the same as previously reports [60,61]. An acidic environment was more favorable for sorbicillinoid production than alkaline conditions, which was similar to the reports on the production of other mycotoxins, such as patulin [50], aflatoxin [62], and trichothecenes [63]. Darkness was more conducive for mycelial growth, but light enhanced the sporulation of *U. virens,* which also observed in other fungal species [23,60]. Light exposure considerably increased the sorbicillinoid content in mycelia, which was different from the study reporting the constitutive hyper production of sorbicillinoids in *T. reesei* ZC121 [64].

The expression of genes participating in secondary metabolism is usually strictly controlled by environmental factors through global regulators, and is regulated at the transcriptional level [24,26]. Recently, we identified the gene cluster responsible for sorbicillinoid biosynthesis; six conserved genes in *sor* BGC and one gene outside the cluster were involved in sorbicillinoid biosynthesis pathway in *U. virens* [22]. The expression levels of most sorbicillinoid-biosynthesis-related genes demonstrated a positive association with sorbicillinoid production with different carbon and nitrogen sources, an ambient pH and light exposure. In particular, UvSorT, an MFS transporter, is responsible for the export and efflux of sorbicillinoids, and its transcriptional levels were up-regulated significantly when using the culture conditions that resulted in the high production of sorbicillinoids. The correlation between UvSorT transcript levels and sorbicillinoid production should be studied in detail. However, it is difficult to reveal the correlation between carbon sources and sorbicillinoid biosynthesis. The possible reason is that there is a correlation between a certain carbon source and other genes’ expression (not *sor* BGC expression). This might explain why the addition of lactose, stachyose, and dextrin in the medium resulted in an increased mycelial radial expansion, as well as why these carbon sources were clustered in the same clade. Conducive carbon and nitrogen sources, an acidic environment and light exposure induced a remarkable up-regulation of *sor* BGC genes and enhanced sorbicillinoid production, in *U. virens*. Expression levels of *CreA*, *AreA*, *PacC* and velvet complex (*VeA/VelB/LaeA*) orthologue genes under different environmental factors in *U. virens* were also investigated (Figure S3). Both *CreA* and *LaeA* had been confirmed to be involved in sorbicillinoid regulation in *T. reesei,* in previous studies. [42,44]. CreA-mediated carbon catabolite repression (CCR) participates in morphology, pathogenicity and SM production [65,66]. Glucose can stimulate high transcript levels of *CreA* in CCR, which was also observed in our study [67,68]. The relative expression levels of *UvAreA* in various nitrogen sources were different, supporting the function of AreA as a nitrogen metabolism regulator, used to confer the ability to use a wide variety of nitrogen sources in fungi [34]. PacC is a pH factor involved in regulation of SM biosynthetic pathways in *Aspergillus* or *Fusarium* species [63]. In *U. virens*, the expression levels of *UvPacC* were higher under alkaline environments, with similar reports for *PePacC*, an activator under acidic conditions, of patulin biosynthesis in *P. expansum* [50]. The velvet complex (*VeA*/*VelB*/*LaeA*) has been implicated as a light-sensitive regulator of SMs in fungi [32]. The function of *UvVeA* in regulating the development and virulence of *U. virens* was revealed recently [69]. *UvLaeA* and *UvVeA* expression levels decreased slightly in the presence of light, revealing that they might mediate light regulation of sorbicillinoids. Considering that these global regulators mediate the regulation of carbon and nitrogen sources, ambient pH and light on sorbicillinoid biosynthesis in *U. virens*, other strategies, such as gene deletion, overexpression and RNA–seq are worth studying in future investigations.

Both *SorR1* and *SorR2,* as the pathway-specific regulator genes in *sor* BGC, have been reported in *P. chrysogenum* and *T. reesei*. *SorR1* acted as a transcriptional activator, while *SorR2* controlled the expression of *SorR1*, and they balanced carbon and secondary metabolism in *T. reesei* [39,44,46,70]. Gene deletion and RT–qPCR confirmed that *UvSorR1* and *UvSorR2* positively participate in the sorbicillinoid biosynthesis regulation, at transcriptional levels. As two backbone genes, *UvSorA* and *UvSorB* were detected with low expression levels, and small amounts of the backbone product sorbicillin still accumulated in the medium with culture of the *ΔUvSorR1* mutant, which was different from *P. chrysogenum* and *T. reesei* [39,70]. Additionally, *UvSorR2* under constant light exposure (LL) was remarkably up-regulated by 86-fold when compared to the constant dark (DD), which supported previous results, in which *SorR2* was considered as a regulator in response to light, and subjected to regulation by photoreceptors [21]. More details are needed to reveal the connection between the sorbicillinoid biosynthesis and environmental factors, global transcription factors and pathway-specific regulators. Our results may provide valuable information for understanding the complex regulation mechanism and network of sorbicillinoid biosynthesis in *U. virens*.

## 5. Conclusions

In summary, this study revealed the effects of carbon and nitrogen sources, ambient pH, and light exposure on mycelial growth, sporulation and sorbicillinoid production in *U. virens*. Among these environmental factors, glucose and sucrose, peptone and tryptone, an ambient pH of 6.0 and darkness were more conducive to mycelial growth. Fructose, beef extract, a pH of 5.0 and light exposure stimulated sporulation. Moreover, glucose and fructose, yeast extract paste and beef extract, acidic conditions and light exposure were favorable for sorbicillinoid production. Sorbicillinoid biosynthesis was mainly regulated at the transcriptional level by different environmental factors, and environmental factors also modulated the expression of global transcription factors. Two pathway-specific regulation genes, *UvSorR1* and *UvSorR2,* as positive regulators, directly regulated sorbicillinoid biosynthesis at the transcriptional level. These findings will provide useful information, in order to better understand the complex and multi-level regulatory network regarding fungal development and sorbicillinoid biosynthesis in *U. virens*, as well as be advantageous in developing effective strategies for controlling fungal diseases and mycotoxin contamination. Other environmental factors, such as the incubation temperature, shaking, aeration, redox status, and metal ions, along with their regulation mechanisms, should be further studied. Investigating gene deletions and overexpressions by phenotypic analysis of relevant regulators to reveal the mechanisms of regulation, also merit further investigation.

## Data Availability

Not applicable.

## Supplementary Materials

The following supporting information can be downloaded at: https://www.mdpi.com/article/10.3390/jof9040390/s1, Figure S1: Effects of ambient pH on the production of main sorbicillinoids in *U. virens*; Figure S2: Comparisons of the colony extension, mycelial growth and sporulation of wild-type strain P1 and gene deletion mutants of *U. virens* cultured on PDA medium at 28 °C for 18 days; Figure S3. Expression of global regulator genes in *U. virens* grown on different carbon and nitrogen sources, ambient pH, and light treatments; Table S1: Fungal strains and plasmids used in this study; Table S2: Primers used in this study; Table S3: Effects of carbon sources on the contents and yields of main sorbicillinoids in *U. virens*; Table S4: Effects of nitrogen sources on the contents and yields of main sorbicillinoids in *U. virens*; Table S5: Effects of ambient pH on contents and yields of main sorbicillinoids in *U. virens*; Table S6: Effects of light exposure on contents and yields of main sorbicillinoids in *U. virens*. The references [57,58,71] cited in Table S1 of the Supplementary Materials are listed in the section References of the text.
