# Supplementary material for: Effects of Carbon, Nitrogen, Ambient pH and Light on Mycelial Growth, Sporulation, Sorbicillinoid Biosynthesis and Related Gene Expression in Ustilaginoidea virens
Source: J Fungi (Basel). 2023 Mar 23;9(4):390. doi: 10.3390/jof9040390 (PMC10142091; doi:10.3390/jof9040390)
Supplement: Supplementary file 1 [file jof-09-00390-s001.zip › jof-2217269-supplementary.pdf]

Supplementary Materials:

Effects of Carbon, Nitrogen, Ambient pH and Light on Mycelial Growth, Sporulation, Sorbicillinoid Biosynthesis and Related Gene Expression in *Ustilaginoidea virens*

Xuping Zhang, Xuwen Hou, Dan Xu, Mengyao Xue, Jiayin Zhang, Jiacheng Wang, Yonglin Yang, Daowan Lai, and Ligang Zhou *

Department of Plant Pathology, College of Plant Protection, China Agricultural University, Beijing 100193, China; zhangxuping5@cau.edu.cn (X.Z.); xwhou@cau.edu.cn (X.H); cauxudan@cau.edu.cn (D.X.); mengyaoxue@cau.edu.cn (M.X); jiayinzhang@cau.edu.cn (J.Z.); yonglinyang@cau.edu.cn (Y.Y.)
* Correspondence: lgzhou@cau.edu.cn; Tel.: +86-10-6273-1199

Contents

Figure S1. Effects of ambient pH on the production of main sorbicillinoids in *U. virens*. (**A**) Chemical structures of trichotetronine, demethylthyltrichodimerol and trichodimerol; (**B**) HPLC profiles of the *U. virens* cultivated in PBGY medium with different ambient pH values. S3

Figure S2. Comparisons of the colony extension, mycelial growth and sporulation of wild-type (WT) strain P1 and gene deletion mutants (Δ*UvSorR1* and Δ*UvSorR2*) of *U. virens* cultured on PDA medium at 28 °C for 18 days. The colony diameter (mm), mycelial biomass (mg) and sporulation (conidia/mm^2) were measured to statistical analysis. The error bars represent the standard deviations, and the asterisks represent significant differences at p < 0.05. All data showed that Δ*UvSorR1* and Δ*UvSorR2* mutants had no significant differences with WT strain. S4

Figure S3. Expressions of global regulator genes in *U. virens* grown on different carbon and nitrogen sources, ambient pH, and light treatments. Data were analyzed using the $2^{-\Delta\Delta Ct}$ method, and normalized using the *β-tubulin* as housekeeping gene. (**A**) Relative expression analysis of *UvCreA* gene under the various carbon sources; (**B**) Relative expression analysis of *UvAreA* gene under the various nitrogen sources; (**C**) Relative expression analysis of *UvPacC* gene under different ambient pH; (**D**) Relative expression analysis of velvet complex (*UvLaeA*, *UvVeA*, and *UvVelB*) under different light exposure treatments. Different letters in each figure mean significant difference according to Duncan's Multiple Range Test (p < 0.05). S5

Table S1. Fungal strains and plasmids used in this study. S6

Table S2. Primers used in this study. S7

Table S3. Effects of carbon sources on contents and yields of main sorbicillinoids (trichotetronine, demethylthyltrichodimerol, trichodimerol) in *U. virens.* S9

Table S4. Effects of nitrogen sources on contents and yields of main sorbicillinoids (trichotetronine, demethylthyltrichodimerol, trichodimerol) in *U. virens*. S9

Table S5. Effects of ambient pH on contents and yields of main sorbicillinoids (trichotetronine, demethylthyltrichodimerol, trichodimerol) in *U. virens*. S10

Table S6. Effects of light exposure on contents and yields of main sorbicillinoids (trichotetronine, demethylthyltrichodimerol, trichodimerol) in *U. virens*. S11

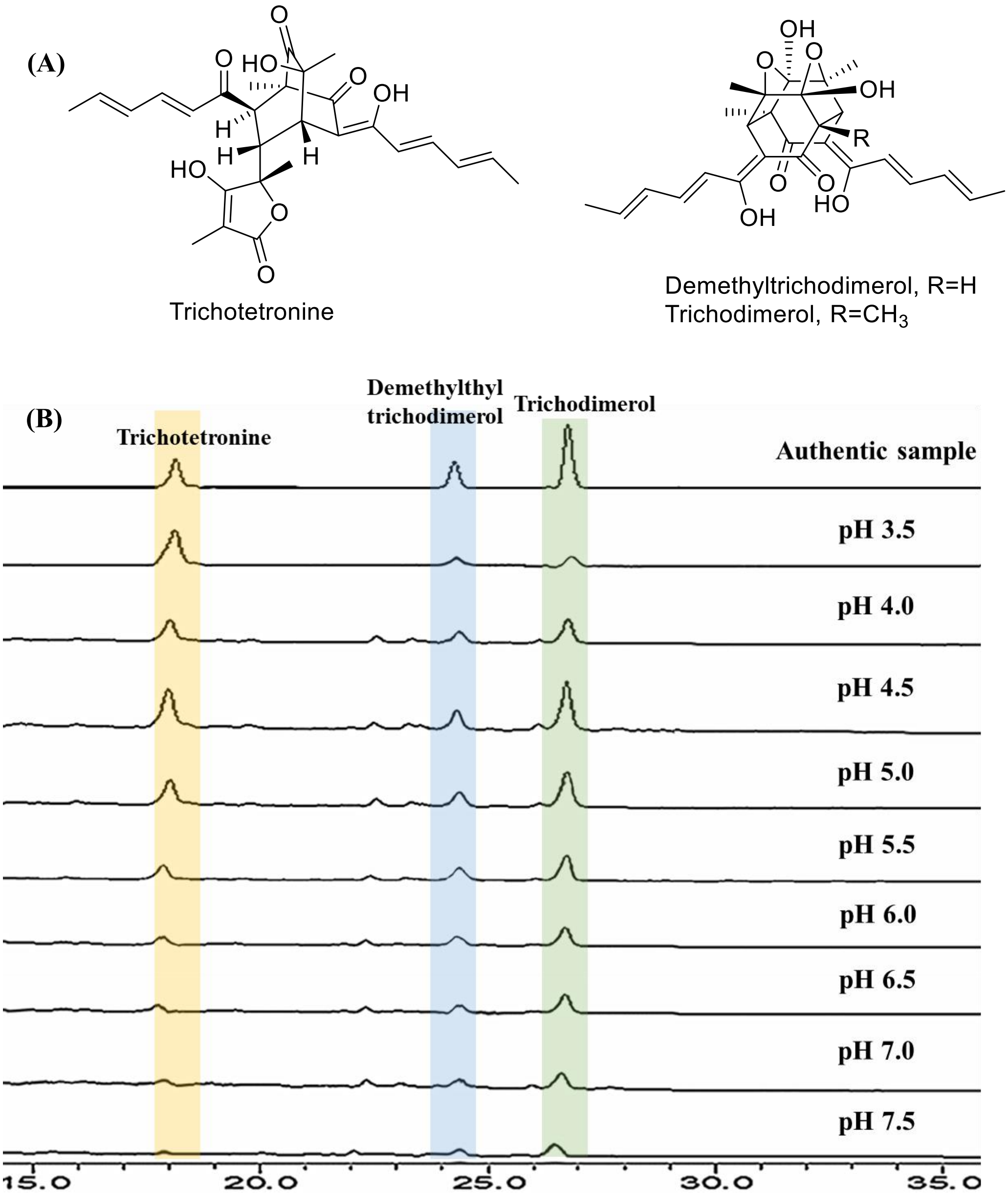

Figure S1. Effects of ambient pH on the production of main sorbicillinoids in *U. virens*. (**A**) Chemical structures of trichotetronine, demethylthyltrichodimerol and trichodimerol; (**B**) HPLC profiles of the *U. virens* cultivated in PBGY medium with different ambient pH values.

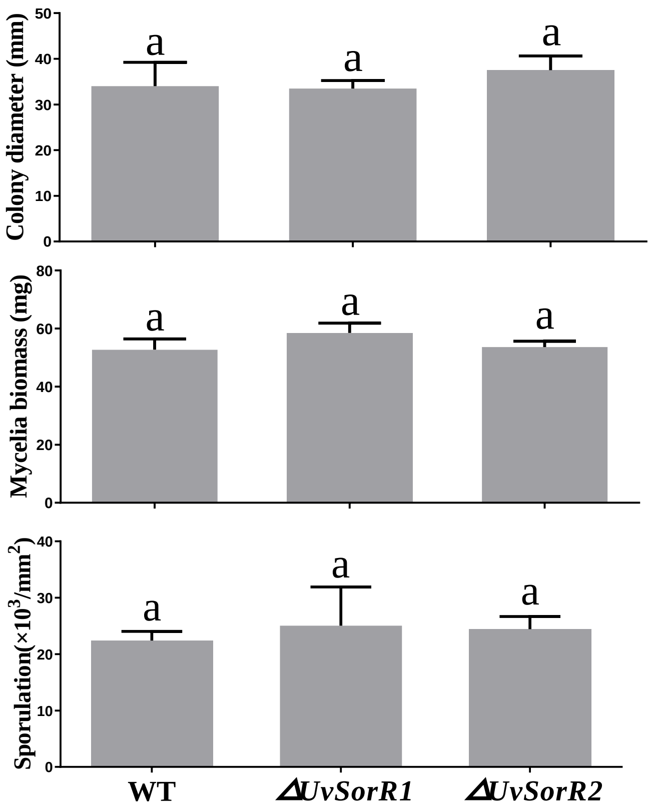

Figure S2. Comparisons of the colony extension, mycelial growth and sporulation of wild-type (WT) strain P1 and gene deletion mutants (Δ*UvSorR1* and Δ*UvSorR2*) of *U. virens* cultured on PDA medium at 28 °C for 18 days. The colony diameter (mm), mycelial biomass (mg) and sporulation (conidia/mm^2) were measured to statistical analysis. The error bars represent the standard deviations, and the asterisks represent significant differences at $p < 0.05$. All data showed that Δ*UvSorR1* and Δ*UvSorR2* mutants had no significant differences with WT strain.

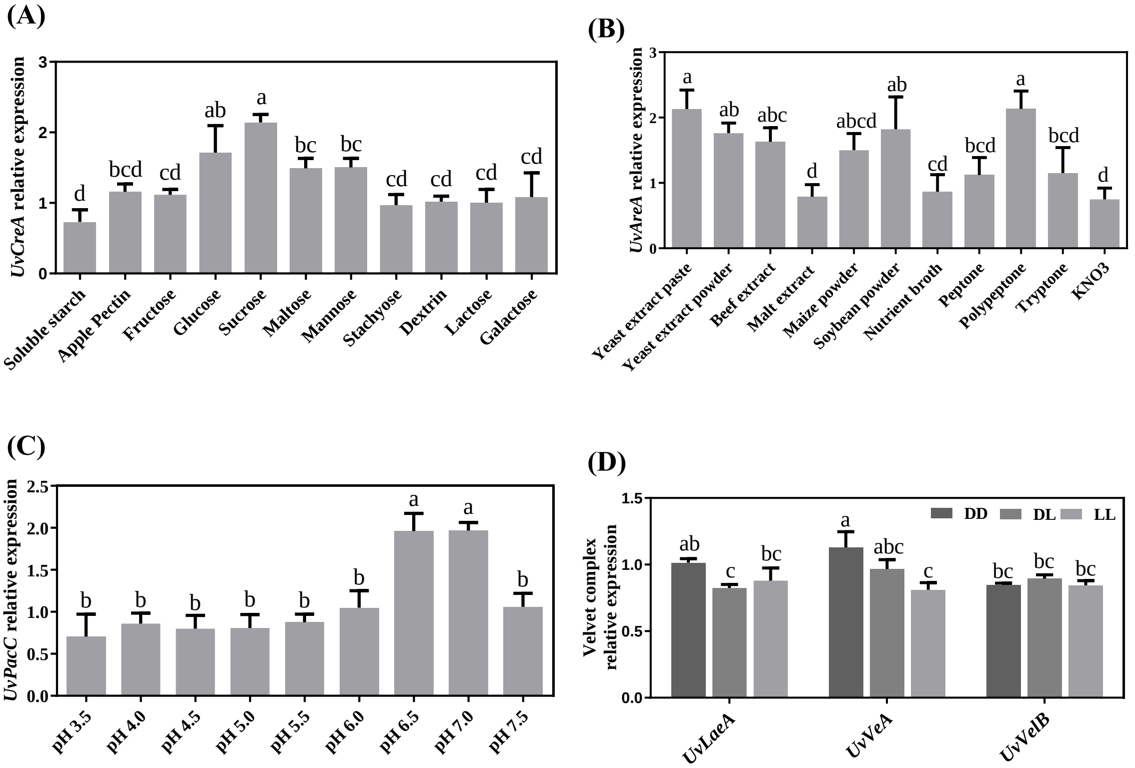

Figure S3. Expressions of global regulator genes in *U. virens* grown on different carbon and nitrogen sources, ambient pH, and light treatments. Data were analyzed using the $2^{-\Delta\Delta Ct}$ method, and normalized using the *β-tubulin* as housekeeping gene. (**A**) Relative expression analysis of *UvCreA* gene under the various carbon sources; (**B**) Relative expression analysis of *UvAreA* gene under the various nitrogen sources; (**C**) Relative expression analysis of *UvPacC* gene under different ambient pH; (**D**) Relative expression analysis of velvet complex (*UvLaeA*, *UvVeA*, and *UvVelB*) under different light exposure treatments. Different letters in each figure mean significant difference according to Duncan's Multiple Range Test ($p < 0.05$).

Table S1. Fungal strains and plasmids used in this study.

Fungal Strain/Plasmid		Description	Ref.
**Fungal strain**	*Ustilaginoidea virens* P1	Wild-type	[57]
	Δ*UvSorR1*	*UvSorR1* deletion mutant of wild type P1	This study
	Δ*UvSorR2*	*UvSorR2* deletion mutant of wild type P1	This study
**Plasmid**	pCas9-tRp-gRNA	Cas9-gRNA vector with the tRNA promoter	[58]
	pFL2	pFL2 containing geneticin-resistance (GenR)	[71]
	pCas9-tRp-UvSorR1	Cas9-gRNA vector with the UvSorR1 spacer	This study
	pCas9-tRp-UvSorR2	Cas9-gRNA vector with the UvSorR2 spacer	This study

Note: This table is supplemental to the experimental procedure.

Table S2. Primers used in this study.

Primer	Oligonucleotide Sequence (5'-3')	Uses
UvSorR1_5F	CGGACCTCGTCATCTTCAT	*UvSorR1*'5 flanks amplification
UvSorR1_5R	CAGATACGGCAGAGAAATCGCAACCTCA AGAGCGAGACATTCAGGGA	
UvSorR1_3F	GTTTAGATTCCAAGTGTCTACTGCTGGCG GAAGAACAACACGACCAT	*UvSorR1*' 3 flanks amplification
UvSorR1_3R	ACGGCAAGATGACAGACAG	
UvSorR1 _F	GCTACGCTTGGCATCCTTG	*UvSorR1* transformant screening
UvSorR1 _R	CGTATTAGCAACCCGAGACC	
KOUvSorR1_5F	TGATGATGAGGCGGTGAAG	*UvSorR1* transformants 5F and 3F screening
KOUvSorR1_3R	TAGCCCATCCTGCCTGACT	
UvSorR1_spacer_F	ACCTAAGAAGGAGAGATCTGACCG	Constructing pCas9-tRp-UvSorR1 vector
UvSorR1_spacer_R	AAACCGGTCAGATCTCTCCTTCTT	
UvSorR2_5F	GAACGCTCGGTTTCCTGTG	*UvSorR2*'5 flanks amplification
UvSorR2_5R	CAGATACGGCAGAGAAATCGCAACCTCG AACGGCAAATCGCAGTAG	
UvSorR2_3F	GTTTAGATTCCAAGTGTCTACTGCTGGCA TGGAGTTACGCCCAGGAG	*UvSorR2*'3 flanks amplification
UvSorR2_3R	GCTGTGCTGAACGCTCTTG	
UvSorR2_F	CACCATCACAGCCACGAAT	*UvSorR2* transformant screening
UvSorR2_R	GCAATGTGGAGGTAATAGACG	
KOUvSorR2_5F	AGTGTCGCAATGGTGGTGAT	*UvSorR2* transformants 5F and 3F screening
KOUvSorR2_3R	TCAGTTCGTCGTAGGTGGC	
UvSorR2_spacer_F	ACCTATATCGCGATCCGTACCGAG	Constructing pCas9-tRp-UvSorR2 vector
UvSorR2_spacer_R	AAACCTCGGTACGGATCGCGATAT	
GEN_F	GAGGTTGCGATTTCTCTGCCGTATCTG	*GenR* amplication from pFL2
GEN_R	GCCAGCAGTAGACACTTGGAATCTAAAC	
855_R	TGTTGGGTTTGAGCTAGGTGGG	Upstream /downstream flanks amplification
856_F	GAATGGTCAAATCAAACTGCTAGATAT	
β-tubulin _F	AGGTTGCGTTGAAGGAGGTT	*U. virens* *β-tubulin* as the control
β-tubulin _R	GAGGTGGAGTTGCCGATAAA	
RT_UvSorA_F	CTTTTCGACGCGGCATTCTT	RT-qPCR for *UvSorA*
RT_UvSorA_R	GCACAAAGACGCCAGTGAAG	
RT_UvSorB_F	GGACCTCCATGTGGTTCCAG	RT-qPCR for *UvSorB*
RT_UvSorB_R	TGATAGCGCTCTTCCCAACG	
RT_UvSorC_F	CGTCTCTGTCCACGTCCTTG	RT-qPCR for *UvSorC*
RT_UvSorC_R	CAGAGGCGTTGGCTGTTTTG	
RT_UvSorD_F	CCATCCACGTTACCCCGATT	RT-qPCR for *UvSorD*
RT_UvSorD_R	CCATGAGCCCTCCATCTTGG	

RT_UvSorR1_F	CAGGGCCTCCACCAAATTCT	RT-qPCR for *UvSorR1*
RT_UvSorR1_R	AGGTGGCTGTTCTCGTCAAG	
RT_UvSorR2_F	GCGGTCGACACAGGAAGATT	RT-PqCR for *UvSorR2*
RT_UvSorR2_R	CGCTGTGGCAGTGCAATTAG	
RT_ UvSorT_F	GGTCTATTGTGTAGCGCCGT	RT-qPCR for *UvSorT*
RT_ UvSorT_R	GAACCGGTCCGTATGTCTCG	
RT-UvLaeA_F	CTGCGAGGTCTGTGGTGAAT	RT-qPCR for *UvLaeA*
RT-UvLaeA_R	CTACCTCCCAACGTGCGATT	
RT-UvVeA_F	GAGTAGTTACGCTGGGGCTC	RT-qPCR for *UvVeA*
RT-UvVeA_R	CCGTACATCAAGTCGGAGCA	
RT-UvVelB_F	TGTAGCATTTGCGTGCGATG	RT-qPCR for *UvVelB*
RT-UvVelB_R	ACCCAGCCGTGATTCAACAT	
RT-UvPacC _F	CAAGATTACGTCCGGTGCCT	RT-qPCR for *UvPacC*
RT-UvPacC _R	AGTTCCACTGGCACGTAAGG	
RT-UvAreA_F	ATTCCCATCAAGCCTCGCAA	RT-qPCR for *UvAreA*
RT-UvAreA_R	CCAGCTGTGTTGCTGTTGAC	
RT-UvCreA_F	GTGAATGGAGGAGGGGCATC	RT-qPCR for *UvCreA*
RT-UvCreA_R	CCCTCCTCACCCACCTTTTC	

Note: This table is supplemental to the experimental procedure.

Table S3. Effects of carbon sources on contents and yields of main sorbicillinoids (trichotetronine, demethylthyltrichodimerol, trichodimerol) in *U. virens*.

Carbon Source	Trichotetronine		Demethylthyltrichodimerol		Trichodimerol		Total Sorbicillinoids	
	Content (mg/g)	Yield (mg/L)	Content (mg/g)	Yield (mg/L)	Content (mg/g)	Yield (mg/L)	Content (mg/g)	Yield(mg/L)
Soluble starch	0.0884±0.0047 g	0.0440±0.0036 e	0.0000±0.0000 h	0.0000±0.0000 f	0.0202±0.0060 d	0.0101±0.0033 g	0.1086±0.0106 f	0.0541±0.0068 e
Apple pectin	1.5433±0.0491 c	2.2503±0.3322 d	0.1257±0.0149 d	0.1820±0.0211 c	0.1263±0.0153 c	0.1824±0.0143 e	1.7952±0.0594 c	2.6147±0.3532 d
Fructose	3.6631±0.2403 a	5.0723±0.5539 b	0.3163±0.0134 b	0.4377±0.0373 b	1.0997±0.0840 a	1.5167±0.0504 a	5.0792±0.1789 a	7.0267±0.5415 a
Glucose	1.8514±0.0524 b	5.8074±0.2287 a	0.0496±0.0095 e	0.1555±0.0293 c	0.2243±0.0218 b	0.7045±0.0821 c	2.1253±0.0768 b	6.6674±0.3243 a
Sucrose	1.4384±0.0775 cd	4.7949±0.4608 bc	0.0444±0.0032 ef	0.1476±0.0088 c	0.1064±0.0043 c	0.3539±0.0127 d	1.5892±0.0795 d	5.2963±0.4791 c
Maltose	1.3233±0.0547 de	4.5765±0.1734 c	0.1534±0.0083 c	0.5310±0.0390 a	0.2284±0.0230 b	0.7880±0.0417 b	1.7050±0.0748 cd	5.8954±0.1863 b
Mannose	0.5929±0.0416 f	1.8978±0.0314 d	0.0187±0.0011 g	0.0602±0.0071 d	0.1201±0.0088 c	0.3848±0.0250 d	0.7317±0.0447 e	2.3429±0.0126 d
Stachyose	0.1372±0.0202 g	0.1999±0.0111 e	0.0395±0.0026 ef	0.0582±0.0070 d	0.0323±0.0023 d	0.0476±0.0067 fg	0.2090±0.0240 f	0.3056±0.0219 e
Dextrin	0.1260±0.0206 g	0.1963±0.0086 e	0.0320±0.0068 fg	0.0510±0.0160 de	0.0563±0.0018 d	0.0888±0.0106 f	0.2143±0.0184 f	0.3361±0.0190 e
Lactose	0.1452±0.0280 g	0.0823±0.0045 e	0.0000±0.0000 h	0.0000±0.0000 f	0.0202±0.0017 c	0.0116±0.0015 g	0.1654±0.0286 f	0.0939±0.0031 e
Galactose	1.2190±0.0669 e	0.2680±0.0144 e	0.3759±0.0126 a	0.0826±0.0010 d	0.1053±0.0115 d	0.0232±0.0028 g	1.7003±0.0817 cd	0.3738±0.0177 e

Note: All values represent mean ± standard deviation; values marked with different letters in each column indicate significant differences ($p < 0.05$).

Table S4. Effects of nitrogen sources on contents and yields of main sorbicillinoids (trichotetronine, demethylthyltrichodimerol, trichodimerol) in *U. virens*.

Nitrogen Source	Trichotetronine		Demethylthyltrichodimerol		Trichodimerol		Total Sorbicillinoids	
	Content (mg/g)	Yield (mg/L)	Content (mg/g)	Yield (mg/L)	Content (mg/g)	Yield (mg/L)	Content (mg/g)	Yield(mg/L)
Yeast extract	1.4102±0.1417 a	7.4642±1.1144 a	0.2954±0.0198 b	0.5634±0.0513 b	0.2954±0.0198 cd	1.5552±0.0221 c	1.8127±0.1412 a	9.5828±1.1479 a
Yeast extract powder	0.0260±0.0013 h	0.1415±0.0077 e	0.0105±0.0010 e	0.0571±0.0051 g	0.0244±0.0043 g	0.1326±0.0224 f	0.0610±0.0060 g	0.3312±0.0308 f
Beef extract	1.1222±0.0827 b	4.9952±0.2952 b	0.1734±0.0105 a	0.7718±0.0209 a	0.3338±0.0154 bc	1.4861±0.0389 c	1.6294±0.1019 b	7.2531±0.3196 b
Malt extract powder	0.5785±0.0727 de	2.0152±0.1082 d	0.0356±0.0035 d	0.1242±0.0036 de	0.1099±0.0083 e	0.3836±0.0028 e	0.7240±0.0844 d	2.5230±0.1133 e
Maize powder	0.6586±0.0147 d	2.1525±0.0192 d	0.0330±0.0034 d	0.1079±0.0080 ef	0.2499±0.0156 d	0.8166±0.0414 d	0.9415±0.0293 c	3.0770±0.0346 de
Soybean powder	0.5404±0.0226 def	1.9873±0.0332 d	0.0413±0.0059 cd	0.1517±0.0195 de	0.1062±0.0066 e	0.3903±0.0138 e	0.6879±0.0324 de	2.5293±0.0504 e
Nutrient broth	0.4531±0.0267 f	2.3228±0.4001 d	0.0150±0.0021 e	0.0760±0.0028 fg	0.0961±0.0071 ef	0.4895±0.0553 e	0.5642±0.0332 e	2.8883±0.4578 de
Peptone	0.9858±0.0182 c	5.7209±0.0996 b	0.0504±0.0059 c	0.2925±0.0336 c	0.5516±0.0180 a	3.2016±0.1219 a	1.5878±0.0209 b	9.2150±0.1521 a
Poly peptone	0.4643±0.0456 ef	2.4848±0.3151 d	0.0308±0.0074 d	0.1619±0.0177 d	0.1442±0.0129 e	0.7728±0.1059 d	0.6392±0.0642 de	3.4195±0.4210 d
Tryptone	0.6018±0.0310 d	3.7598±0.6001 c	0.0437±0.0065 cd	0.2689±0.0153 c	0.3556±0.0855 b	2.1761±0.3363 b	1.0010±0.0734 c	6.2048±0.3780 c
KNO_3	0.3008±0.0109 g	0.8939±0.0124 e	0.0146±0.0033 e	0.0433±0.0094 g	0.0462±0.0027 fg	0.1374±0.0122 f	0.3615±0.0112 f	1.0745±0.0209 f

Note: All values represent mean ± standard deviation; values marked with different letters in each column indicate significant differences ($p < 0.05$).

Table S5. Effects of ambient pH on contents and yields of main sorbicillinoids (trichotetronine, demethylthyltrichodimerol, trichodimerol) in *U. virens*.

**Ambient pH**	**Trichotetronine**		**Demethylthyltrichodimerol**		**Trichodimerol**		**Total Sorbicillinoids**	
	Content (mg/g)	Yield (mg/L)	Content (mg/g)	Yield (mg/L)	Content (mg/g)	Yield (mg/L)	Content (mg/g)	Yield(mg/L)
pH 3.5	1.8940±0.2754 a	3.3235±1.0248 a	0.0193±0.0033 c	0.0327±0.0042 d	0.0756±0.0050 e	0.1345±0.0499 f	1.9889±0.2721 a	3.4907±1.0718 bc
pH 4.0	1.4207±0.2375 b	3.4230±0.8297 b	0.0643±0.0100 a	0.1529±0.0180 cd	0.2675±0.0297 b	0.6367±0.0484 de	1.7525±0.2084 ab	4.2126±0.8146 bc
pH 4.5	1.0746±0.0253 c	5.7990±0.3861 c	0.0528±0.0050 ab	0.2853±0.0361 bc	0.4101±0.0153 a	2.2105±0.0913 a	1.5375±0.0338 b	8.2949±0.4921 a
pH 5.0	0.8891±0.0274 c	6.4376±0.2469 a	0.0477±0.0067 ab	0.3455±0.0523 ab	0.2008±0.0174 c	1.4543±0.1422 b	1.1375±0.0277 c	8.2374±0.3194 a
pH 5.5	0.4322±0.1116 d	3.1214±0.7718 b	0.0562±0.0283 ab	0.4162±0.2246 ab	0.1185±0.0276 d	0.8546±0.1796 c	0.6069±0.0858 d	4.3922±0.6022 b
pH 6.0	0.2483±0.0402 de	1.9917±0.1714 c	0.0606±0.0083 ab	0.4865±0.0283 a	0.0827±0.0117 e	0.6722±0.1323 cd	0.3916±0.0381 de	3.1504±0.0512 cd
pH 6.5	0.1919±0.0075 def	1.4139±0.0823 c	0.0422±0.0026 b	0.3111±0.0264 bc	0.0619±0.0017 e	0.4561±0.0082 e	0.2960±0.0085 ef	2.1811±0.1066 d
pH 7.0	0.0774±0.0064 e	0.5007±0.0401 d	0.0159±0.0013 c	0.1029±0.0084 d	0.0283±0.0024 f	0.1833±0.0159 f	0.1217±0.0078 fg	0.7869±0.0484 e
pH 7.5	0.0255±0.0038 e	0.0680±0.0087 d	0.0098±0.0002 c	0.0261±0.0012 d	0.0118±0.0013 f	0.0315±0.0039 f	0.0471±0.0051 g	0.1257±0.0123 e

Note: All values represent mean ± standard deviation; values marked with different letters in each column indicate significant differences ($p < 0.05$).

Table S6. Effects of light exposure on contents and yields of main sorbicillinoids (trichotetronine, demethylthyltrichodimerol, trichodimerol) in *U. virens*.

Light	Trichotetronine		Demethylthyltrichodimerol		Trichodimerol		Total Sorbicillinoids	
	Content (mg/g)	Yield (mg/L)	Content (mg/g)	Yield (mg/L)	Content (mg/g)	Yield (mg/L)	Content (mg/g)	Yield(mg/L)
DD	0.9162±0.0638 c	4.9952±0.2952 a	0.1416±0.0076 b	0.7718±0.0209 b	0.2540±0.0427 c	1.3831±0.2109 a	1.3118±0.1085 c	7.1501±0.4864 ab
DL	1.6071±0.3253 b	5.8267±0.9588 a	0.2996±0.0112 a	1.0911±0.0214 a	0.3692±0.0669 b	1.3382±0.1743 a	2.2759±0.4002 b	8.2560±1.1168 a
LL	3.7917±0.4554 a	4.9648±0.1285 a	0.3681±0.0722 a	0.4829±0.0786 c	0.9557±0.0488 a	1.2616±0.1442 a	5.1154±0.5027 a	6.7093±0.1320 b

Note: DD, 24 h darkness/day; LL, 24 h light/day; DL,12 h darkness-12 h light/day: All values represent mean ± standard deviation. The values marked with different letters in each column indicate significant differences ($p < 0.05$).
